# L-Carnitine and Acetyl-L-Carnitine: Potential Novel Biomarkers for Major Depressive Disorder

**DOI:** 10.3389/fpsyt.2021.671151

**Published:** 2021-09-30

**Authors:** Li-Juan Nie, Jun Liang, Feng Shan, Bao-Shi Wang, Yuan-Yuan Mu, Xie-Hai Zhou, Qing-Rong Xia

**Affiliations:** ^1^Affiliated Psychological Hospital of Anhui Medical University, Hefei, China; ^2^Department of Pharmacy, Hefei Fourth People's Hospital, Hefei, China; ^3^Psychopharmacology Research Laboratory, Anhui Mental Health Center, Hefei, China; ^4^Anhui Clinical Research Center for Mental Diseases, Hefei, China

**Keywords:** biomarker, acetyl-l-carnitine, diagnosis, depression, L-Carnitine

## Abstract

The lack of biomarkers greatly limits the diagnosis and treatment of major depressive disorder (MDD). Endogenous L-carnitine (LC) and its derivative acetyl-L-carnitine (ALC) play antidepressant roles by improving brain energy metabolism, regulating neurotransmitters and neural plasticity. The levels of ALC in people and rodents with depression are significantly reduced. It is necessary to determine whether serum LC and ALC might be used as novel biomarkers for the diagnosis of MDD. Here, ultra-high performance liquid chromatography-tandem mass spectrometry (UPLC-MS/MS) was used to determine the concentration of LC and ALC in the serum of healthy controls and patients with MDD; among the latter, in patients who were responsive (effective group) and non-responsive (ineffective group) after 2 weeks of treatment. The diagnostic value of serum LC and ALC for MDD was assessed. Compared with healthy controls, the serum LC and ALC concentrations in patients with MDD were significantly decreased (*P* < 0.001). Pearson correlation analysis shows that the HDRS-24 score was negatively associated with serum ALC (*r* = −0.325, *P* = 0.007). Receiver operating characteristic (ROC) analysis revealed an area under the curve (AUC) of 0.801 with 83.1% sensitivity and 66.3% specificity for LC, and an AUC of 0.898 with 88.8% sensitivity and 76.4% specificity for ALC, differentiating patients with MDD from healthy controls. Furthermore, the concentration of LC and ALC in patients with depression was significantly increased in the effective treatment group, and no significant change was observed in the ineffective treatment group. These results suggest that serum LC and ALC may be novel biomarkers for the diagnosis of MDD.

## Introduction

Major depressive disorder (MDD) is a common mental disorder that is the leading cause of disability around the World (1, 2). According to a recent survey, the lifetime and 12-month prevalence rates of depression in China are 3.4 and 2.1%, respectively (3). The pathogenesis of the disease is relatively complex, which is generally considered to be related to genetics, sex, neuroendocrine, psychosocial environment, immunity, intestinal microorganisms, and other factors (4–6). At present, clinical diagnosis is mainly based on the description of symptoms by the patient, mental state examination, and clinical behavior observation, lacking objective diagnosis indicators (7–9), which greatly increases the incorrect diagnosis. The effect of drug therapy is not obvious in some patients, which is also related to incorrect diagnosis (10). Therefore, it is of great significance to explore the pathogenesis of depression and search for biomarkers for the diagnosis and diversified treatment of clinical depression.

Studies have shown that neuroplasticity impairment may be the significant pathophysiological mechanism of depression (11, 12). In addition, studies demonstrated that acetyl-L-carnitine (ALC) has multiple functions related to neuroplasticity (13) and is an antidepressant substance with significant potential. ALC is a natural form of L-carnitine (LC). High concentrations of carnitines either free carnitine or as acylcarnitines including ALC, are present in biological tissues and cells (14). ALC is an endogenous compound, and it is mainly present in brain, kidney, and liver. Its main physiological function is to promote coenzyme A to enter into the mitochondria, thus promoting β-oxidation of long-chain fatty acids (15). In addition to improving mitochondrial function and energy, and enhancing antioxidant activity (16), ALC has been shown to be effective in a variety of neuropsychiatric disorders, such as Alzheimer’s disease (17, 18), attention deficit hyperactivity disorder (19), depression (20) and depressive symptoms in the course of fibromyalgia (21), multiple sclerosis (22), and alcohol dependence (23). In addition, animal studies (24–26) have shown that, in rodents with depression-like characteristics, ALC levels were significantly reduced, ALC supplementation can induce rapid and durable antidepressant-like effects through the epigenetic mechanism of histone acetylation, antidepressant responses were seen after 3 d that also last for 14 d after drug withdrawal. The clinical research results (27, 28) showed that serum ALC levels in patients with severe depression are significantly lower than those in healthy people, and ALC supplementation significantly reduced depressive symptoms.

Acetyl-CoA and LC in the body are synthesized into ALC by carnitine acetyltransferase catalyzed. LC is also involved in the β-oxidation pathway. The deficit of LC can result in impairment of β-oxidation, which in turn leads to the consumption of acetyl-CoA, and ultimately leads to the reduction of ALC production (29). There is also a certain relationship between LC and depression. Studies (30, 31) have reported that serum free carnitine levels were significantly reduced in patients undergoing hemodialysis treatment, and free carnitine levels with a low baseline level are independently associated with the severity of depression in male patients. Clinical studies (31, 32) have shown that L-carnitine supplementation can improve the depression state of male uremic patients and cancer patients.

Therefore, we aimed to apply UPLC-MS/MS method to determine the concentrations of LC and ALC in serum, compare the differences in the levels of LC and ALC between MDD patients and healthy controls; analyze the levels of LC and ALC correlation with the degree of depression; and study the role of ALC and LC levels in the onset and diagnosis of depression.

## Materials and Methods

### Participants

A total of 261 individuals with a first depression episode (according to the Diagnostic and Statistical Manual for Psychiatric Disorders, fourth edition) hospitalized at the Anhui Mental Health Center from November 2018 to October 2020, were scanned in this study, 89 patients with MDD were selected. The inclusion criteria were as follows: the age ranged from 18 to 70 years; a primary diagnosis of MDD in a current major depressive episode; the reduction rate of the 24-item Hamilton Depression-Rating Scale (HDRS-24) score in the treatment-effective group was ≥50% and the reduction rate of HDRS-24 score in the treatment-ineffective group was <50% (33). A significant improvement in patients’ depressive symptoms was observed after treatment in the effective-treatment group. The exclusion criteria were as follows: alcohol and drug abusers; use of immunomodulators, various antidepressants, or lithium salts in the last half year; history of serious heart, liver, kidney, and other serious body diseases, metabolic diseases, and infectious diseases such as cold and fever in the last 2 weeks; combination with other mental illness or nervous system disease history; pregnancy and lactation. The healthy control (HC) individuals were recruited from the Anhui Medical University Health Checkup Center. HC were free of lifelong mental and major illnesses and metabolic diseases. The subjects had no active infections and systemic diseases confirmed by the medical history at the time of the study evaluation. Blood samples were collected from the vein through standard techniques before the evaluation meeting and all subjects were fasting. Tubes with a 5 mL capacity were used to collect the blood. Samples were centrifuged at 3,000 r/min for 5 min at 4°C and the serum was collected. The serum was stored at −80°C for further use. According to the principles of the Declaration of Helsinki, all subjects provided informed written consent before participating. This study was approved by the Ethics Committee of the Anhui Provincial Mental Health Center. All our researches have been adhered to standard biosecurity and institutional safety procedures.

Clinical assessment consisted of a physical examination, including measures of height, weight, body mass index (BMI), and biochemical indexes. Additional information was collected, including demographics, current drug use, Hamilton Anxiety Scale (HAMA) score and HDRS-24 score. Regarding drug use, 89 patients with a first depression episode did not take any antidepressants while they participated in the study. In addition, we evaluated the LC and ALC levels of 55 patients after treatment. Blood samples were collected through the vein 2–6 weeks after treatment, and were divided into treatment-effective group and treatment-ineffective group according to the HDRS-24 score reduction rate.

### UPLC-MS/MS Determination of LC and ALC

A simple, rapid, and selective UPLC-MS/MS method for the determination of LC and ALC in human serum was developed. The UPLC-MS/MS instrumentation was composed of a Waters Acquity ultra performance liquid chromatography (UPLC) I Class Binary Solvent Manager, Acquity UPLC Sample Manager-FTN coupled to a Waters Xevo TQ-S mass spectrometer (Waters, Massachusetts, USA). Acetyl-L-carnitine-d_3_ (ALC- d_3_) was selected as the internal standard (IS). After protein precipitation with acetonitrile-water (1 mL, 2:1, v/v), the analytes and IS were separated on a 2.5 μm Waters XSelect HSS T3 C18 column through gradient elution with methanol-water (containing 0.01% aqueous ammonia) as the mobile phase at a flow rate of 0.2 mL/min. Analytes were detected with multiple reaction monitoring using a positive scan mode with electrospray ionization (ESI). The ratios of signal intensities for the transitions m/z 162.10 > 102.97 (LC), m/z 204.14 > 85.03 (ALC), and 207.19 > 85.03 (ALC-d_3_) were converted to concentration using a calibration curve. ALC hydrochloride (>98% purity) was purchased from Sigma-Aldrich (St. Louis, MO, USA). L-carnitine·HCl (>98% purity) and ALC-d_3_·HCl (>98% purity) were purchased from Toronto Research Chemicals Inc. (TRC, North York, Canada). All the other analytical reagents and solvents were above chromatographic purity level. A quality control was conducted for each experiment to ensure the accuracy of the results.

### Statistical Analysis

All statistical analyses were conducted using the SPSS version 17.0 (SPSS, Chicago, Illinois, USA). Student’s *t*-test and χ^2^ analysis were used to compare the continuous and classified demography and clinical characteristics of HC and patients with MDD. The serum LC and ALC concentrations of all the individuals in the HC and MDD groups were compared with an independent-sample *t*-test and the serum LC and ALC concentrations of MDD before and after treatment were compared with a paired-sample *t*-test. The relation between HDRS-24 scores and the other variables were analyzed with Pearson correlation tests and the independent relationships were determined by multivariate linear regression analysis. The receiver operating characteristic (ROC) curve analysis was used to determine the area under the curve (AUC) and cut-off values of serum LC and ALC. *P* < 0.05 was considered statistically significant. Unless otherwise specified, the data are expressed as mean ± SD.

## Results

### LC and ALC Levels Differ Between Healthy Controls and Patients With MDD

**Figure 1:**
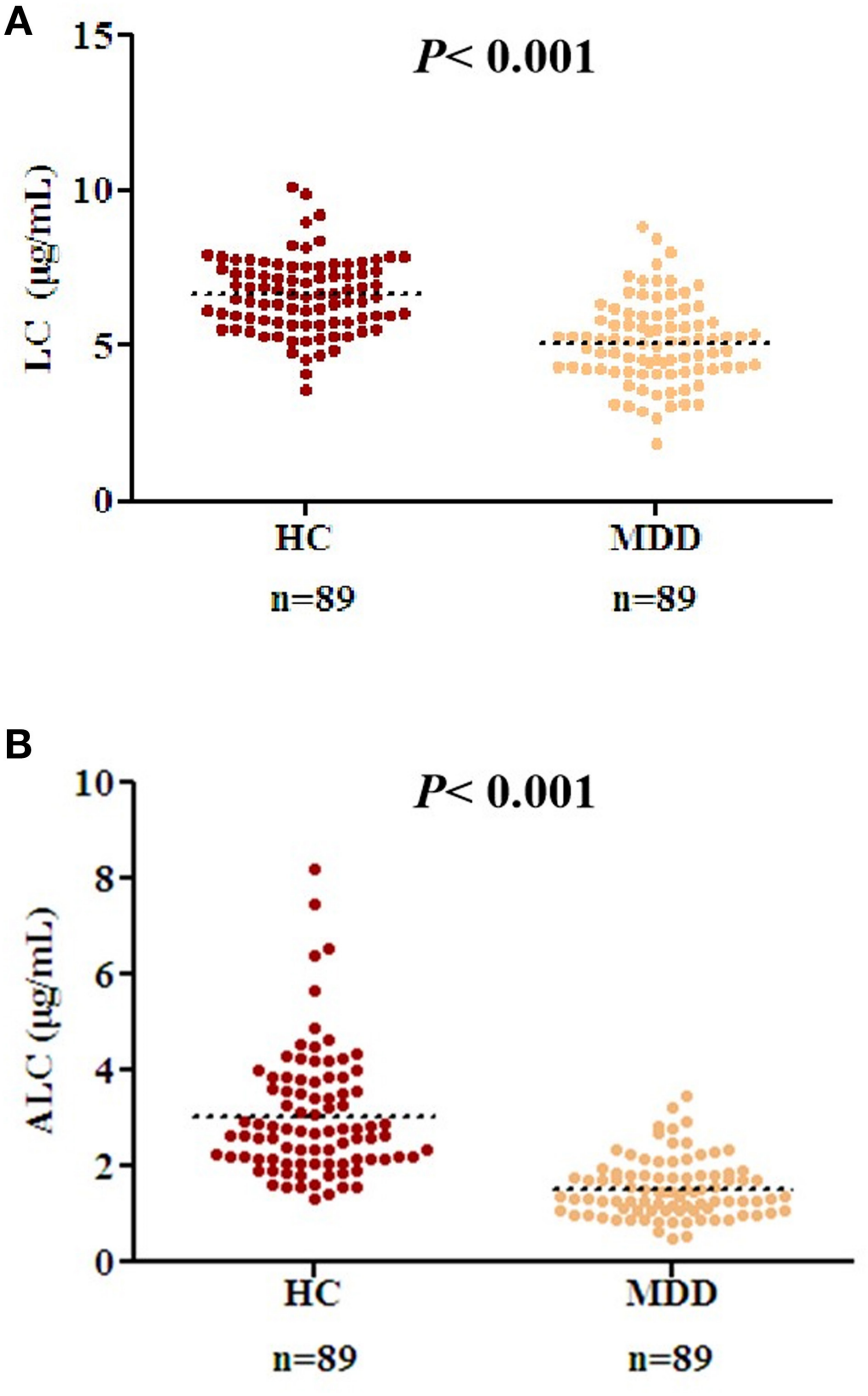
Decreased acetyl-L-carnitine (ALC) and L-carnitine (LC) levels in patients with MDD compared to those in the HC group. **(A)** Serum LC concentrations in HC and patients with MDD; **(B)** ALC concentrations in HC and patients with MDD. *P* < 0.001 was considered statistically significant. Dashed bars indicate group mean.

There were no differences in age, BMI, gender, and other demographic characteristics between the HC (*n* = 89) and patients with MDD (*n* = 89) (Table 1). All the patients were in an acute depressive episode during study participation. The level of LC in the MDD group was lower than that in the HC (Figure 1A, *P* < 0.001, *t* = 8.01, effect size = 1.20, Power =1.00, HC: 6.64 μg/mL ± 1.20, MDD: 5.12 μg/mL ± 1.33). Similarly, the level of ALC in the MDD group was significantly lower than that in the HC group (Figure 1B, *P* < 0.001, *t* = 9.93, effect size = 1.49, Power = 1.00, HC: 3.05 μg/mL ± 1.30, MDD: 1.54 μg/mL ± 0.60).

**Table 1:** Comparison of age, gender, and BMI between the MDD and the HC groups (mean ± SD).

**Variables**	**MDD group**	**HC group**	**Test** **(***t*** or χ^**2**^)**	***P*** **-value**
Age	36.92 ± 16.34	37.00 ± 15.22	−0.033	0.974
Gender (female/male)	57/32	58/31	0.025^a^	0.875
BMI (kg/m^2^)	21.58 ± 3.51	22.30 ± 3.49	−1.378	0.170

a *Indicates χ^2^ score. P < 0.05 was considered statistically significant*.

**Figure 2:**
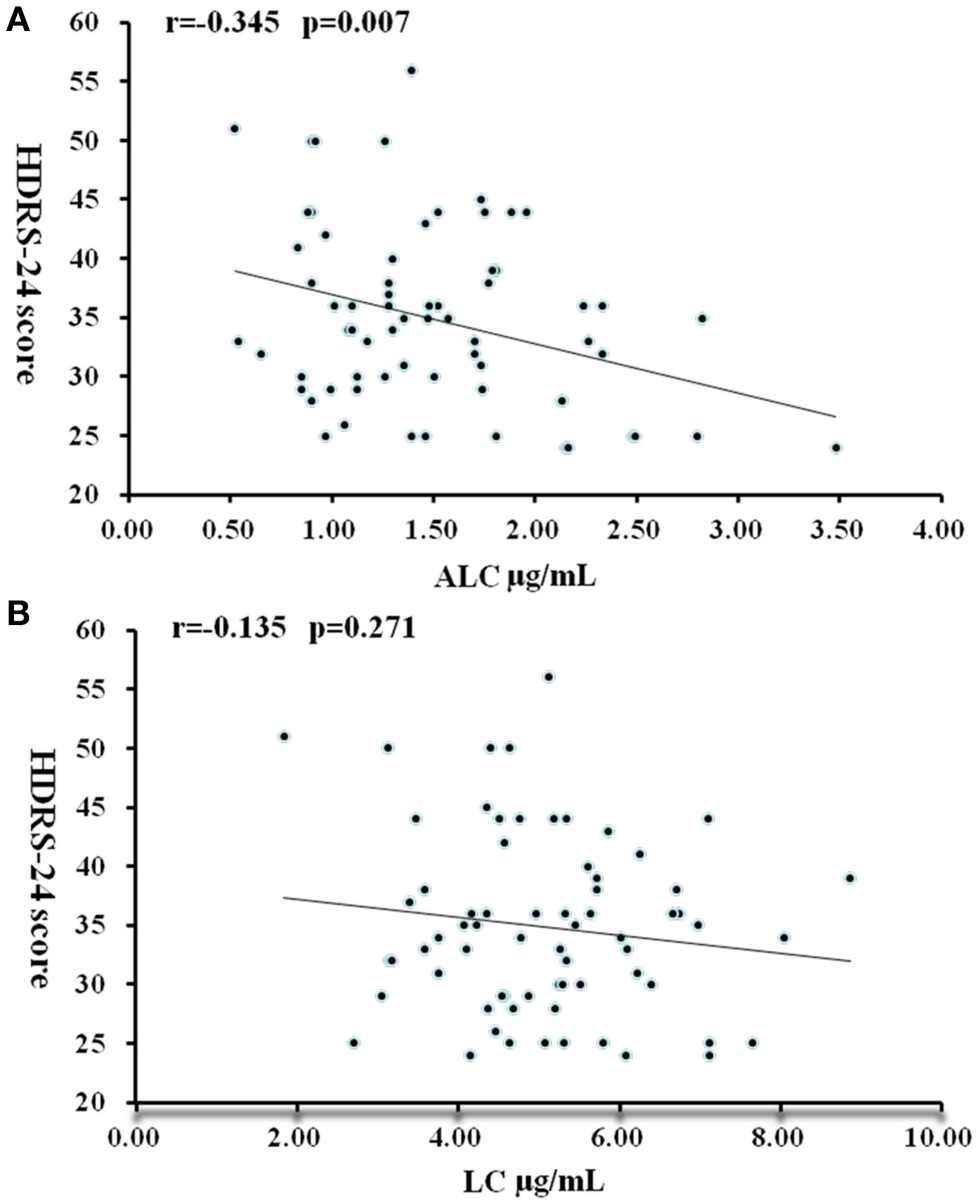
Correlation between HDRS-24 scores and serum concentrations of ALC **(A)** and LC **(B)** in patients with MDD. *P* < 0.05 was considered statistically significant.

In patients with moderate to severe MDD (HDRS-24 score > 24), ALC levels were significantly negatively correlated with severity scores: the higher the severity, the lower the ALC concentrations (r = −0.325, *P* = 0.007) (Figure 2). In the multiple regression analysis controlling for BMI value, gender, and age, this relationship was still significant (*t* = −2.79, *P* = 0.007). No correlation was found between LC concentration and HDRS-24 scores (*r* = −0.135, *P* = 0.271).

**Figure 3:**
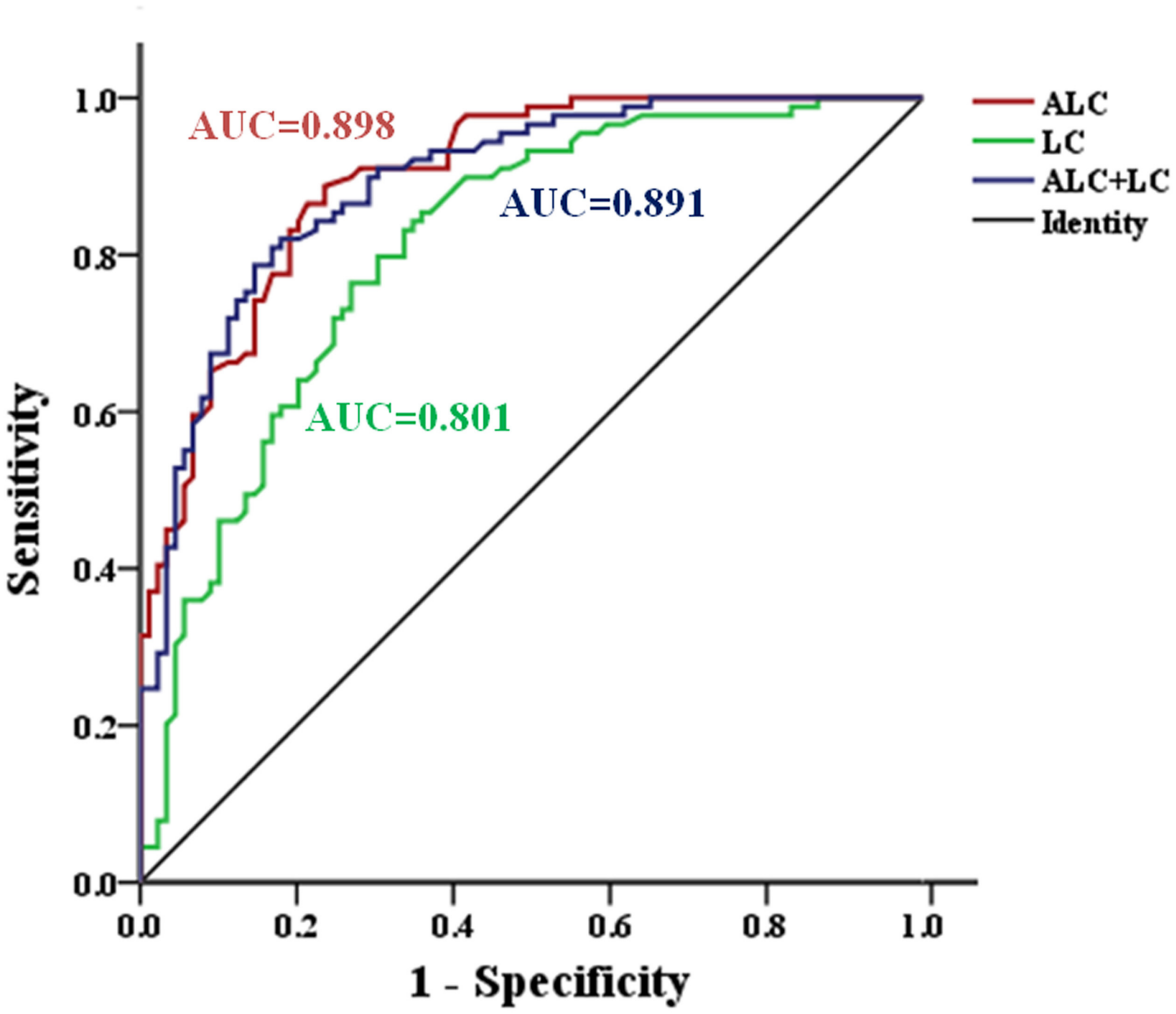
ROC curve for serum ALC and LC in the identification of patients with MDD. AUC, area under curve; MDD, major depressive disorder; ROC, Receiver operating characteristic.

In addition, the ROC curve analysis showed potential diagnostic values of serum ALC and LC (Figure 3). The AUC of LC and ALC were 0.801 and 0.898, respectively. When the critical value of LC was 5.52 μg/mL, the sensitivity and specificity of MDD patients and HC were 83.1 and 66.3%, respectively. At the ALC critical concentration of 1.835 μg/mL, the sensitivity was 88.8%, and the specificity was 76.4%. When the LC and ALC results were considered together, the ROC analysis showed that the AUC of patients with MDD and HC was 0.891, the sensitivity was 82.0%, and the specificity was 82.0%.

### ALC and LC Levels Differ Before and After Treatment

Since the collection of blood samples after treatment was not considered at the beginning of the study, as well as the impact of the new coronary pneumonia epidemic and the time of hospitalization for <2 weeks, blood samples of 34 patients were not collected after treatment. Finally, from June 2019 to October 2020, sera were collected from 38 patients with effective treatment and 17 patients with ineffective treatment. There were no differences in age, BMI, gender, HDRS-24 scores before treatment, treatment days and other demographic characteristics between the effective-treatment and ineffective-treatment groups, there was significant difference in the reduction rate of HDRS-24 scores and HDRS-24 scores after treatment between the two groups (Table 2). Additionally, the results of covariance analysis showed that the difference of reduction rate of HDRS-24 scores between effective group and ineffective group was not affected by treatment days and the HDRS-24 scores before treatment (*F* = 17.26, *P* < 0.001). In the effective-treatment group, the LC levels were significantly higher after treatment compared to those before treatment (*t* = −3.53, *P* = 0.001, size effect = 0.53) (Table 3). Similarly, the ALC levels were significantly higher after treatment than those before treatment (*t* = −2.05, *P* = 0.047, size effect = 0.32) (Table 3). The LC and ALC levels in the ineffective-treatment group were not significantly increased after treatment (detailed results are shown in Table 3).

**Table 2:** Comparison of age, gender, BMI and the reduction rate of HDRS-24 scores between effective-treatment and ineffective-treatment groups (mean ± SD).

**Variables**	**Effective-treatment group**	**Ineffective-treatment group**	**Test** **(***t*** or χ^**2**^)**	***P*** **-value**
Age	37.08 ± 16.91	38.82 ± 22.86	−0.316	0.753
Gender (female/male)	23/15	10/7	0.014^a^	1.000
BMI (kg/m^2^)	21.12 ± 3.45	20.19 ± 3.62	0.916	0.364
**HDRS-24 scores**				
Before treatment	34.11 ± 8.63	31.59 ± 9.98	0.953	0.345
After treatment	7.42 ± 5.77	20.88 ± 6.02	−7.888	<0.001
Treatment days	23.42 ± 10.24	19.35 ± 6.69	1.752	0.087
Reduction rate of HDRS-24 scores (%)	79.08 ± 15.06	31.59 ± 15.74	10.657	<0.001

a *Indicates χ^2^ score*.

*P < 0.05 was considered statistically significant*.

**Table 3:** Comparison of serum concentrations of LC (μg/mL) and ALC (μg/mL) before and after treatment (mean ± SD).

	**Variables**	**Before treatment**	**After treatment**	**Statistics**	***P*** **-value**	**Effect size**	**Power**
Effective treatment group (*n* = 38)	LC	5.11 ± 1.45	5.98 ± 1.76	−3.530	0.001^**^	0.53	0.99
	ALC	1.79 ± 0.69	2.06 ± 0.91	−2.050	0.047^*^	0.32	0.86
	LC+ALC	6.90 ± 1.78	8.04 ± 2.22	−3.607	0.001^**^	0.56	0.99
Ineffective- treatment group (*n* = 17)	LC	5.25 ± 1.23	5.22 ± 1.38	0.111	0.913		
	ALC	2.01 ± 0.54	1.70 ± 0.58	1.643	0.120		
	LC+ALC	7.26 ± 1.35	6.92 ± 1.69	1.165	0.261		

** *P < 0.01*,

* *P < 0.05 were considered statistically significant*.

## Discussion

LC is a non-essential amino acid derived from the essential amino acids lysine and methionine, whose balance is maintained through dietary intake and endogenous formation followed by renal excretion (29). In the brain, LC and its derivative ALC are present in high concentration, reducing nerve damage by regulating mitochondrial permeability and preventing excitatory toxicity (34). LC and ALC may play antidepressant roles by improving brain energy metabolism (35), regulating neurotransmitters (36, 37), and neural plasticity (38–40). In agreement with the results obtained using a depression rodent model and clinical MDD samples studied by Nasca et al. (27), the ALC level in patients with MDD was significantly lower than that of the age- and gender-matched HC group. Similarly, consistent with their findings, we also found a negative correlation between ALC level and HDRS-24 score. It is worth noting that a previous article reported positive correlation between peripheral and central nervous system ALC concentrations (14). Based on the ROC analysis, the serum ALC cut-off point of 1.835 μg/mL showed a 88.8% sensitivity and a 76.4% specificity, indicating that serum ALC has a superior diagnostic value (AUC = 0.898) in MDD. These results suggest that ALC may be a diagnostic marker of depression.

In contrast to the above-mentioned clinical study (27), we found that LC levels in MDD patients were significantly lower than those in HC group. This difference may result from race, sample size, and eating habits. ROC curve analysis showed that the AUC of LC was 0.801. These results showed that LC was closely related to depression and had good diagnostic value for MDD. Pearson correlation analysis showed that HDRS-24 scores had no correlation with serum LC concentration, which may be because ~75% of human carnitine comes from exogenous sources (mainly from meat and dairy products), and L-carnitine does not cross the blood-brain barrier directly to exert antidepressant effects like acetyl-L-carnitine. Carnitine acetyltransferase catalyzes the synthesis of ALC from acetyl-CoA (CoA) and LC on the inner membrane of mitochondria, and enters the mitochondrial matrix through carnitine/acetylcarnitine acyltransferase. Carnitine penetrates into the mitochondrial matrix, and carnitine acetyltransferase converts ALC into LC and acetyl-CoA (41). Because LC and ALC are transformed into each other in the body, the LC and ALC contents were positively correlated (MDD: *r* = 0.273, *P* = 0.01, HC: *r* = 0.293, *P* = 0.005), many studies have officially confirmed that LC and ALC have a synergistic effect on the treatment of diseases (42, 43), and ROC analysis showed that AUC of patients with MDD and HC was 0.891 when LC and ALC were considered together. LC and ALC may therefore be considered potential biomarkers of depression simultaneously. In order to avoid the effect of the drug on the baseline carnitine content, the patients enrolled in the group were all patients with first-onset depression who had not undergone drug treatment. Further studies are needed to assess whether reduced LC and ALC levels in MDD patients are sensitive to unhealthy lifestyle choices such as lack of exercise, poor diet, smoking, drugs and lack of sleep.

Many clinical studies (44, 45) have confirmed that, compared with established antidepressants, ALC can significantly reduce depressive symptoms and have similar effects. However, no studies have reported the changes of LC and ALC levels in patients after antidepressant treatment. To verify the correlation between the levels of LC and ALC and the outcome of depression, we compared the changes of LC and ALC in patients with depression before and after treatment. In the treatment group where the HDRS-24 score reduction rate was >50%, the content of LC and ALC increased significantly after antidepressant treatment. The concentrations of LC and ALC in the ineffective treatment group did not change significantly. Perhaps due to the small sample size, we did not find a difference in ALC and LC levels between the effective and ineffective groups, but the changes in ALC and LC before and after treatment were significantly different between the two groups. These results indicate that LC and ALC levels are related to the occurrence and efficacy of depression, but further studies are needed to prove it. We did not intervene in clinical drug treatment. Due to the large number of therapeutic drugs and the small sample size, we did not classify and describe the therapeutic drugs in detail, nor did we consider the impact of different drugs on the level of carnitine after treatment. Next, we hope to further expand the sample size to compare the effects of different types of antidepressants on the LC and ALC concentrations in patients with depression and the effects of different baseline LC and ALC concentrations on the efficacy of antidepressants.

This study has several limitations. First, the study was a single-center study with a small sample size; the samples after treatment were not grouped in detail. Second, the effects of diet, exercise, sleep, and other external factors on LC and ALC concentrations were not considered. Third, the effects of different drugs on the LC and ALC concentrations after treatment were not considered.

In conclusion, the detection of serum LC and ALC may prove to have clinical value in the diagnosis of MDD. Multicenter and longitudinal studies are clearly needed to validate the potential of LC and ALC as novel biomarkers for MDD.

## Data Availability Statement

The raw data supporting the conclusions of this article will be made available by the authors, without undue reservation.

## Ethics Statement

The studies involving human participants were reviewed and approved by Ethics Committee of the Anhui Provincial Mental Health Center. The patients/participants provided their written informed consent to participate in this study.

## Author Contributions

Q-RX, JL, and L-JN designed the study, wrote the protocol, and performed the statistical analysis. Q-RX carried out the study and collected important background information. L-JN performed the experiments and drafted the manuscript. FS and B-SW carried out the concepts, design, definition of intellectual content, literature search, data acquisition, data analysis, and manuscript preparation. Y-YM and X-HZ provided assistance for data analysis and performed the experiments. All authors have read and approved the final manuscript.

## Funding

This project was supported by the Anhui Medical University (Grant No. 2019xkj201), the Hefei Health Applied Medicine (Grant No. hwk2019yb011), and the Hefei Sixth-cycle Key Medical Specialty.

## Conflict of Interest

The authors declare that the research was conducted in the absence of any commercial or financial relationships that could be construed as a potential conflict of interest.

## Publisher's Note

All claims expressed in this article are solely those of the authors and do not necessarily represent those of their affiliated organizations, or those of the publisher, the editors and the reviewers. Any product that may be evaluated in this article, or claim that may be made by its manufacturer, is not guaranteed or endorsed by the publisher.
